# A Core Outcome Set for the Benefits and Adverse Events of Bariatric and Metabolic Surgery: The BARIACT Project

**DOI:** 10.1371/journal.pmed.1002187

**Published:** 2016-11-29

**Authors:** Karen D. Coulman, James Hopkins, Sara T. Brookes, Katy Chalmers, Barry Main, Amanda Owen-Smith, Robert C. Andrews, James Byrne, Jenny L. Donovan, Graziella Mazza, Barnaby C. Reeves, Chris A. Rogers, Janice L. Thompson, Richard Welbourn, Sarah Wordsworth, Jane M. Blazeby, BARIACT working group

**Affiliations:** 1 School of Social and Community Medicine, University of Bristol, Bristol, United Kingdom; 2 Department of Upper GI and Bariatric Surgery, Taunton and Somerset NHS Foundation Trust, Taunton, Somerset, United Kingdom; 3 University of Exeter Medical School, Medical Research, Exeter, United Kingdom; 4 Department of Diabetes and Endocrinology, Taunton and Somerset NHS Foundation Trust, Taunton, Somerset, United Kingdom; 5 Department of General Surgery, University Hospital Southampton NHS Foundation Trust, Southampton, United Kingdom; 6 NIHR CLAHRC West, University Hospitals Bristol NHS Foundation Trust, Bristol, United Kingdom; 7 Clinical Trials and Evaluation Unit, School of Clinical Sciences, University of Bristol, Bristol, United Kingdom; 8 University of Birmingham, School of Sport, Exercise & Rehabilitation Sciences, Edgbaston, Birmingham, United Kingdom; 9 Nuffield Department of Population Health, University of Oxford, Oxford, United Kingdom; 10 Division of Surgery, Head & Neck, University Hospitals Bristol NHS Foundation Trust, Bristol, United Kingdom

## Abstract

**Background:**

Bariatric and metabolic surgery is used as a treatment for patients with severe and complex obesity. However, there is a need to improve outcome selection and reporting in bariatric surgery trials. A Core Outcome Set (COS), an agreed minimum set of outcomes reported in all studies of a specific condition, may achieve this. Here, we present the development of a COS for BARIAtric and metabolic surgery Clinical Trials—the BARIACT Study.

**Methods and Findings:**

Outcomes identified from systematic reviews and patient interviews informed a questionnaire survey. Patients and health professionals were surveyed three times and asked to rate the importance of each item on a 1–9 scale. Delphi methods provided anonymised feedback to participants. Items not meeting predefined criteria were discarded between rounds. Remaining items were discussed at consensus meetings, held separately with patients and professionals, where the COS was agreed. Data sources identified 2,990 outcomes, which were used to develop a 130-item questionnaire. Round 1 response rates were moderate but subsequently improved to above 75% for other rounds. After rounds 2 and 3, 81 and 14 items were discarded, respectively, leaving 35 items for discussion at consensus meetings. The final COS included nine items: “weight,” “diabetes status,” “cardiovascular risk,” “overall quality of life (QOL),” “mortality,” “technical complications of the specific operation,” “any re-operation/re-intervention,” “dysphagia/regurgitation,” and “micronutrient status.” The main limitation of this study was that it was based in the United Kingdom only.

**Conclusions:**

The COS is recommended to be used as a minimum in all trials of bariatric and metabolic surgery. Adoption of the COS will improve data synthesis and the value of research data. Future work will establish methods for the measurement of the outcomes in the COS.

## Abstract 2

Karen Coulman and colleagues present a core outcome set developed by patients and health professionals for BARIAtric and metabolic surgery Clinical Trials (BARIACT).

## Introduction

The worldwide prevalence of obesity has more than doubled since 1980 and is associated with an increased risk of comorbidities, such as type 2 diabetes, and premature death [1]. Surgery is the most effective treatment for patients with severe and complex obesity (body mass index ≥40 or between 35 and 40 with another significant comorbidity that could be improved by weight loss) [2–4]. Common operations undertaken include the Roux-en-Y gastric bypass, the sleeve gastrectomy, and the adjustable gastric band [2,3,5]. Each have different risks and outcome trajectories [2,3,6,7]. Understanding the relative differences between interventions needs data from well-designed and conducted randomised controlled trials (RCTs) to inform decision-making. However, a Cochrane review found that trials were limited by a lack of consistency in outcome reporting, which hampered cross-study comparison and meta-analysis [3]. This review called for the development of a Core Outcome Set (COS) to improve the consistency of outcomes in future trials [3].

A COS is an agreed minimum set of outcomes to be measured and reported in all studies of a particular disease or condition [8]. A COS is not meant to be restrictive, rather the minimum that should be reported [9]. The uptake and use of a COS can help to reduce the heterogeneity of outcomes reported across trials and reduce outcome reporting bias—the selective reporting of some outcomes from those that were originally measured in a study, on the basis of their results [8,10]. A COS can thus improve the quality of the data available to undertake meta-analyses and inform clinical decision-making [11]. The aim of this study was to develop a COS for bariatric and metabolic surgery, including outcomes relating to both the effectiveness and the safety of the surgery (the BARIACT project) for use in future effectiveness trials.

## Methods

### Ethics Statement

Ethical approval from Southwest-Frenchay Research Ethics Committee (reference 11/SW/0248) was obtained.

**Fig 1:**
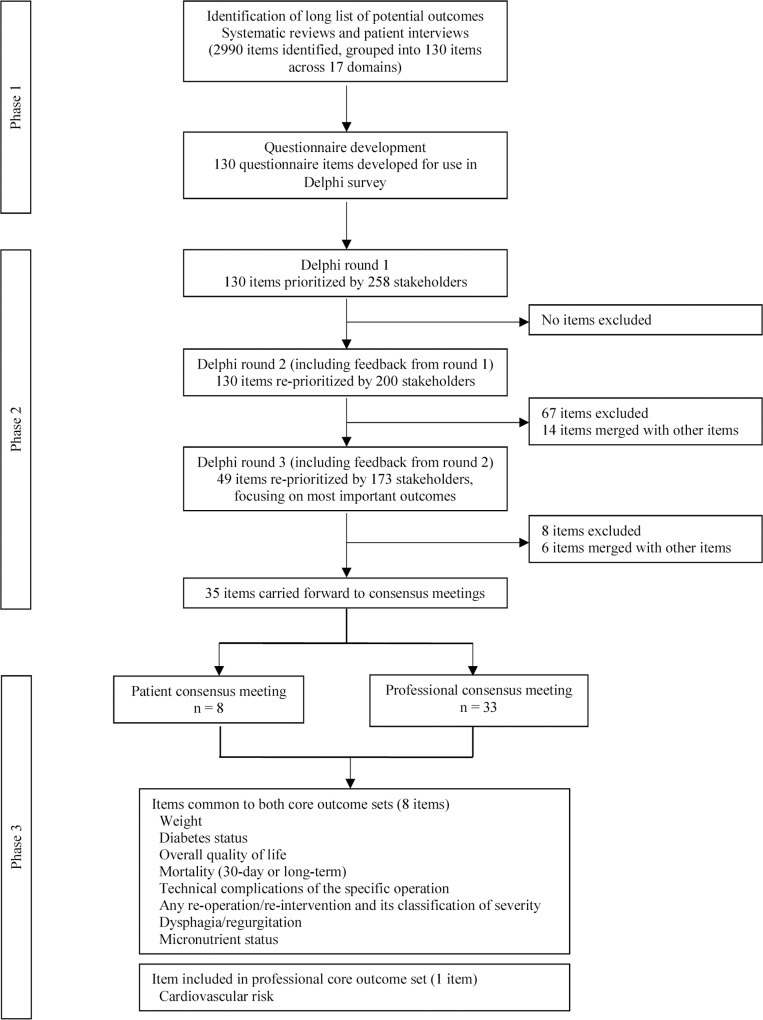
Summary of the development of a COS for bariatric and metabolic surgery.

Development of the COS involved three phases: (1) the generation of a comprehensive list of outcomes and a questionnaire; (2) a Delphi survey involving three rounds to gain consensus as to which outcomes are most important; and (3) patient and professional consensus meetings to agree a final COS. These phases are summarised in Fig 1 and as a table in the supporting information (S1 Table). The project was registered with the COMET (Core Outcome Measures in Effectiveness Trials) Initiative [12,13]. In reporting the development of this COS, we have adhered to the COS-STAR (Core Outcome Set-STAndards for Reporting) Statement (S2 Table) [14].

### Phase 1: Generation of a Comprehensive List of Outcomes and a Questionnaire

A comprehensive list of outcomes of bariatric surgery was informed by literature reviews including qualitative research studies [15–18]. These were supplemented with outcomes elicited from semi-structured interviews with patients [17,18]. All outcomes were independently mapped into health domains by at least two researchers (including expert health professionals and methodologists) [19]. A health domain was defined as a broad class of outcome; for example, the domain “obesity-related disease” included diabetes, hypertension, dyslipidaemia, cardiovascular risk, obstructive sleep apnoea, and joint disease. The final list of domains and outcomes was used to develop a questionnaire, with each outcome forming an individual item and domains forming section headings. Items were written in lay terms with medical terms in brackets to optimise understanding. Further detail on the methodology for this phase of the research has previously been reported [20].

### Phase 2: Delphi Questionnaire Surveys

To ensure the resulting COS was patient-centred, both specialist health professionals involved in the care of bariatric surgery patients and patients who had undergone bariatric surgery were invited to participate in the consensus process. Health professionals (surgeons, nurses, dietitians, psychologists, physicians, and anaesthetists) were identified through professional societies (the British Obesity and Metabolic Surgery Society, the Association of Physicians Specialising in Obesity UK, The Society for Obesity and Bariatric Anaesthesia, the British Psychological Society, and an informal list of bariatric clinical psychologists) and participation in the By-Band-Sleeve Study (a pragmatic RCT comparing gastric bypass, gastric banding, and sleeve gastrectomy) [21]. Individuals were invited to participate by post or email from their Society and were sent an initial questionnaire. Patients who had undergone bariatric surgery in the previous five years at two hospitals participating in the pilot phase of the By-Band-Sleeve Study were purposively sampled (based on gender, type of surgery, and time since surgery) and invited to participate. Patients returning a signed consent form were posted the questionnaire. Non-responding health professionals and patients providing consent but not returning the questionnaire were sent one reminder. In the absence of agreed methodology to determine a sample size for Delphi surveys, the target sample was 100 professionals and 100 patients [22,23].

The Delphi process consisted of three sequential rounds of questionnaires with the same group of participants. Those that completed a questionnaire in round 1 were eligible to participate in round 2, and those that completed round 2 were eligible to participate in round 3. In each questionnaire, participants were asked to rate the importance of each item from 1 (not important) to 9 (extremely important). Responses were summarised and fed back (anonymously) in subsequent rounds. Participants received their own scores, the median score of the overall patient group, and the median score assigned by all health professionals for each item. For professionals, scores were further broken down with the median scores presented for their own peer group, other health professionals, and patients.

All items were retained between rounds 1 and 2. At the end of rounds 2 and 3, items were only retained if they met prespecified criteria (see “Statistical Analyses” section). Further consideration was given by the research team to whether any remaining items could be merged. Items retained at the end of round 3 were considered at the consensus meetings.

### Phase 3: Face-to-Face Consensus Meetings

Consensus meetings were held separately with patients and professionals to ensure that meetings were not dominated by professionals’ views. Meetings were held in Bristol, UK in October and November 2015. Participants completing all three questionnaires were invited to attend, in addition to professional members of the By-Band-Sleeve Study group.

Retained items and median scores for the patient and professional groups were presented and participants asked to vote “Yes” (this item should be included in the COS), “No” (this item should not be included), or “Unsure” using anonymised keypad voting [24]. Item wording was shortened and simplified for the consensus meetings to allow for ease of reading on Microsoft Powerpoint slides, with verbal clarification as needed. Item wording used for patient and professional consensus meetings is provided as supporting information (S3 Table). Voting results for each item were presented immediately in the form of a histogram. Items were retained or dropped when consensus was reached (see “Statistical Analyses” section). Discussion and further rounds of voting, restricting the options to “Yes” or “No,” were undertaken until consensus was reached on all items. All items retained from both meetings were included in the final COS.

### Statistical Analyses

Analyses were undertaken using STATA 13 [25]. After each Delphi round the median score for each item was calculated for patients and professionals and each professional sub-group; median scores were presented as feedback in the subsequent round (round 3 presented in the consensus meetings). For merged items, participants’ scores were calculated as the mean of the individual items’ scores, and group scores were calculated as the mean of the individual items’ median scores.

At the end of rounds 2 and 3, the percentage of participants who rated each item 8 or 9 was calculated, and items were retained if they were scored 8 or 9 by at least 70% of respondents. These criteria were considered separately for patients and professionals, and items were retained if they met these criteria. Items discussed at the consensus meetings were retained if at least 70% of participants voted “Yes”; items were discarded if at least 70% voted “No.”

## Results

### Phase 1

The literature and interviews yielded 2,990 outcomes which were categorised into 17 domains, forming a 130-item questionnaire [20].

### Phase 2

Four hundred fifty-nine professionals were invited, of which 168 (36.6%) returned the questionnaire. The round 2 denominator was reduced to 157 due to the researchers being unable to send questionnaires to 11 professionals (five had not provided contact details in round 1, four were on maternity leave, and two had moved away). 76.4% (120/157) and 85.0% (102/120) completed rounds 2 and 3, respectively. Participating health professionals included 81 (48.2%) surgeons, 33 (19.6%) dietitians, 24 (14.3%) specialist nurses, 12 (7.1%) bariatric physicians, 10 (6.0%) psychologists, three (1.8%) anaesthetists, three (1.8%) GPs, one (0.6%) physiotherapist, and one (0.6%) “other” health professional. The majority (160, 95.2%) of professionals were from the UK, two were from the Republic of Ireland, one was from Belgium, and five did not specify their country.

Of the 465 patients invited to participate, 112 (24.1%) consented. Of these, 90 (80.4%) completed the round 1 questionnaire (56 from centre 1 and 34 from centre 2). One patient withdrew after round 1. 89.9% (80/89), and 88.8% (71/80) completed rounds 2 and 3, respectively. Patients were 65.6% female and had a mean age of 54.4 years (standard deviation [SD] of 9.6 years). The majority (95.6%) were “White British.” Fifty-eight (64.4%) underwent a Roux-en-Y gastric bypass, 21 (23.3%) an adjustable gastric band, six (6.7%) a sleeve gastrectomy, two (2.2%) more than one type of surgery, one (1.1%) “another” type of surgery, and two (2.2%) were awaiting surgery. The mean time since surgery was 3.5 years (SD 2.1 years).

In round 1, 33 items were classed as “very important.” More details are available elsewhere [20]. After providing feedback in round 2, 57 items were classed as “very important.” These were retained for round 3, as well as six “borderline” items (≥ 65% of either patients or professionals rated these items 8–9), which had been highlighted as very important by patients in the qualitative interviews, which informed the initial list of outcomes (Table 1). The remaining 67 items were not carried forward to round 3. Fourteen of the 63 retained items were merged with other items, leading to 49 items on the round 3 questionnaire (Table 1). The rounds 2 and 3 professional and patient questionnaires are provided as supporting information (S1–S4 Questionnaire). The round 1 questionnaires are available elsewhere [20].

**Table 1:** Items retained for round 3.

		% rating item 8–9 in round 2	
	Item details (*n* = 63)	Patients (*n* = 80)	HCPs (*n* = 120)
1	Intra-operative organ injury	78.8	62.2
2	Intra-abdominal abscess	76.3	68.1
3	Wound infection or dehiscence	71.3	35.3
4	Septicaemia	87.5	85.7
5	Gastro-intestinal bleeding^a^	72.2	67.8
6	Intra-abdominal bleeding^a^	71.3	78.2
7	Staple line bleed^a^	78.8	77.3
8	Gastric fistula^b^	86.1	89.0
9	Anastomotic leak^b^	91.3	92.4
10	Bowel stricture	80.0	79.7
11	Anastomotic ulceration	76.3	67.2
12	Band infection^c^	78.7	84.9
13	Band erosion^c^	84.0	94.9
14	Band revisions^c^	74.7	78.8
15	Port revisions^d^	70.7	61.3
16	Port infection^d^	73.3	67.2
17	Port malfunction^d^	73.3	41.5
18	Band slippage	82.7	94.9
19	Internal hernia	78.5	89.8
20	Adhesional obstruction	71.8	49.2
21	Requirement for ventilation	73.4	62.7
22	Ischaemic/coronary heart disease^e^	77.2	41.5
23	Arrhythmia^e^	71.8	21.2
24	Venous thromboembolism	84.8	79.7
25	Cerebrovascular accident	83.5	57.6
26	Renal failure	81.0	49.2
27	Perioperative mortality^f^	92.4	94.9
28	In hospital mortality^f^	91.1	96.6
29	≤30-day mortality^f^	91.1	94.8
30	>30-day mortality	87.3	89.7
31	Dysphagia/regurgitation	65.8	73.3
32	Vitamin levels^g^	67.1	85.8
33	Mineral levels^g^	55.7	80.8
34	Readmission rates	64.6	79.2
35	Weight^h^	62.0	92.4
36	Body mass index^h^	53.2	86.4
37	Hypertension	87.5	84.7
38	Cardiovascular risk	88.8	77.1
39	Diabetes	92.5	98.3
40	Dyslipidaemia	70.9	85.6
41	Obstructive sleep apnoea	84.8	90.8
42	Joint disease	86.8	72.9
43	Being able to carry out usual activities	73.4	81.7
44	Mobility	82.3	78.3
45	Fitness	73.4	45.8
46	Being able to accomplish work tasks, or to take up work	75.9	82.5
47	Feeling in control of weight and appearance	77.2	59.7
48	Excess skin or skin folds following weight loss*	69.6	52.1
49	Having a healthy/balanced eating pattern	77.2	73.9
50	Being able to stop eating when feeling full	81.0	72.3
51	Feeling satisfied and confident with one’s body^i^	70.9	59.7
52	Self-esteem and self-confidence^i^	73.4	58.8
53	Depression	69.6	70.6
54	Anxiety*	65.8	66.4
55	Suicidal thoughts	64.6	73.9
56	Other addictive behaviours*	58.2	68.9
57	Overall quality of sleep	77.2	47.9
58	Relationship with partner/spouse^j^ *	61.0	45.0
59	Relationship with children^j^ *	67.9	54.2
60	Relationship with friends^j^ *	44.3	22.5
61	Feeling able to live a “normal” life	81.0	71.7
62	Feeling in control of health and well-being	79.7	63.0
63	Having a positive outlook on life and expectations for the future	84.8	65.8

HCPs = Healthcare professionals.

Items with superscripts ^a-j^ were merged to create the following single items for round 3.

^a^ “Intra-abdominal bleeding/gastrointestinal bleeding/staple line bleed.”

^b^ “Anastomotic leak/gastric fistula.”

^c^ “Band infection, erosion, and revisions.”

^d^ “Port malfunction/revisions/infection.”

^e^ “Angina/myocardial infarction/arrhythmia.”

^f^ “Death (during the operation or within 30 days of surgery).”

^g^ “Micronutrient levels.”

^h^ Items merged and used to create two new items, “reduction in weight” and “maintaining weight loss/preventing weight re-gain.”

^i^ “Self-esteem and self-confidence.”

^j^ “Relationship with partner/spouse, friends, and/or ability to care for children.”

* Borderline items (at least 65% of either patients or health professionals rated these items 8–9 in round 2) kept in for round 3.

After round 3, 41 items were classed as “very important” by either group and were retained for the meetings (Table 2). As 41 was a large number of items to vote on at a meeting, items were scrutinised by the research team. Six were merged, reducing the number of items to 35 (Table 2). Three other items (“leaks, fistulas, strictures, and ulcerations at anastomosis,” “mortality (30-day or long-term),” and “improvement in diabetes”) rated 8 or 9 by at least 90% of either group were considered to be extremely important and therefore were not discussed further but included in the COS. The merged item “weight” (including weight reduction/maintenance) was also included in the final COS, being highlighted as very important by patients in the qualitative interviews that informed the initial comprehensive list of outcomes. Thus, the total number of items to be voted on at the consensus meetings was 31. The ratings of all questionnaire items for rounds 1, 2, and 3 are provided in the supporting information (S4 and S5 Tables).

**Table 2:** Items brought forward to the consensus meetings.

	% rating item 8–9 in round 3	
Item details (*n* = 41)	Patients (*n* = 71)	HCPs (*n* = 102)
Intra-operative organ injury	77.5	55.9
Intra-abdominal abscess	78.9	61.8
Septicaemia	88.7	82.4
Bleeding problems—includes intra-abdominal, gastrointestinal, and staple line bleeding	80.3	78.4
Anastomotic leak/gastric fistula^a^ *	91.6	90.2
Bowel stricture^a^ *	83.1	67.7
Anastomotic ulceration^a^ *	78.9	52.9
Band infection, erosion, and revisions^b^	84.4	84.3
Band slippage^b^	85.9	88.1
Port problems	79.7	58.8
Internal hernia	82.9	81.4
Needing to go to intensive care unit for ventilation	73.2	48.5
Cardiac problems due to surgery	71.8	31.7
Venous thromboembolism	83.1	77.2
Stroke	84.5	59.4
Renal failure	77.5	37.6
≤30-day mortality^c^ *	90.1	94.1
>30-day mortality^c^ *	90.1	86.3
Dysphagia/regurgitation	70.4	57.8
Problems with micronutrient levels	70.4	67.7
Readmission rates	73.2	72.6
Reduction in weight^d^ *	78.6	88.1
Maintaining weight loss/preventing weight re-gain^d^ *	87.1	89.1
Feeling in control of weight and appearance^d^ *	77.5	50.0
Reduction in hypertension	81.4	74.3
Reduction in cardiovascular risk	84.3	70.0
Improvement in diabetes*	87.1	96.0
Reduction in dyslipidaemia	72.9	64.0
Reduction in obstructive sleep apnoea	84.3	77.2
Improvement in joint disease	81.4	61.0
Ability to carry out usual activities	81.7	67.7
Improved mobility	87.3	75.5
Ability to accomplish work tasks, or to take up work	71.8	66.7
Having a healthy/balanced eating pattern	73.2	63.7
Ability to stop eating when feeling full	83.1	59.8
Improved self-esteem and self-confidence	83.1	68.3
Improvement in depression	74.7	58.4
Reduction in anxiety	73.2	48.5
Feeling able to live a “normal” life	84.3	65.7
Feeling in control of health and well-being	84.5	60.8
Having a positive outlook on life and expectations for the future	87.3	55.9

HCPs = Healthcare professionals.

^a^ Merged to create one item, “Leaks, fistulas, strictures, and ulcerations at anastomosis.”

^b^ Merged to create one item, “Gastric band problems.”

^c^ Merged to create one item, “Mortality (30-day or long-term).”

^d^ Merged to create one item, “Weight.”

* Included as a definite in the final COS and not voted on at consensus meetings.

### Phase 3: Face-to-Face Consensus Meetings

Thirty-seven patients and 46 professionals indicated an interest in attending a consensus meeting. Of these, eight patients and one partner attended the patient meeting. Five were female, with a mean age of 55 years (SD 9.8 years). Seven had undergone a Roux-en-Y gastric bypass, and one had undergone an adjustable gastric band. Their mean time since surgery was 4.3 years (SD 1.9 years).

Thirty-three professionals attended the professional meeting. This included 14 (42.4%) surgeons, 10 (30.3%) specialist nurses, four (12.1%) dietitians, three (9.1%) bariatric physicians, one (3.0%) psychologist, and one (3.0%) “other” health professional. All except one attendee (Australia) were from the UK.

At the consensus meetings, the four pre-agreed items were presented and the remaining 31 voted on. Tables 3 and 4 show the results of the voting and discussion.

**Table 3:** Results of the patient consensus meeting.

	Number of patients				
	(out of 8) voting (%)				
Item	Item In	Unsure	Item Out	Initial views	Final decision
Diabetes status	Definitely in COS—not voted on				Retain item
Mortality (30-day or long-term)	Definitely in COS—not voted on				Retain item
Leaks, fistulas, strictures, and ulcerations at anastomosis	Definitely in COS—not voted on				Retain “leaks, fistulas, strictures, and ulcers at anastomosis/gastric band problems”^a^
Weight	Definitely in COS—not voted on				Retain item
Hypertension	3 (37.5)	2 (25.0)	3 (37.5)	Unsure	Voted out
Cardiovascular risk	4 (50.0)	1 (12.5)	3 (37.5)	Unsure	Voted out
Dyslipidaemia	0 (0.0)	1 (12.5)	7 (87.5)	Out	Voted out
Obstructive sleep apnoea	1 (12.5)	0 (0.0)	7 (87.5)	Out	Voted out
Joint disease	3 (37.5)	3 (37.5)	2 (25.0)	Unsure	Voted out
Ability to carry out usual activities	5 (62.5)	1 (12.5)	2 (25.0)	Unsure	Retain “mobility/ability to carry out usual activities/living a normal life”^a^
Mobility	6 (75.0)	1 (12.5)	1 (12.5)	In	Retain “mobility/ability to carry out usual activities/living a normal life”^a^
Ability to do your work, or to take up work	6 (75.0)	1 (12.5)	1 (12.5)	In	Retain item
Having a healthy/balanced eating pattern	5 (62.5)	0 (0.0)	3 (37.5)	Unsure	Retain “feeling in control”^a^
Ability to stop eating when feeling full	2 (25.0)	3 (37.5)	3 (37.5)	Unsure	Retain “feeling in control”^a^
Self-esteem and self-confidence	7 (87.5)	0 (0.0)	1 (12.5)	In	Retain “self-esteem and self-confidence/depression/having a positive outlook on life and expectations for the future”^a^
Depression	2 (25.0)	2 (25.0)	4 (50.0)	Unsure	Retain “self-esteem and self-confidence/depression/having a positive outlook on life and expectations for the future”^a^
Anxiety	0 (0.0)	2 (25.0)	6 (75.0)	Out	Voted out
Feeling able to live a “normal” life	5 (62.5)	2 (25.0)	1 (12.5)	Unsure	Retain “mobility/ability to carry out usual activities/living a normal life”^a^
Feeling in control of health and well-being	5 (62.5)	3 (37.5)	0 (0.0)	Unsure	Retain “feeling in control”^a^
Having a positive outlook on life and expectations for the future	5 (62.5)	0 (0.0)	3 (37.5)	Unsure	Retain “self-esteem and self-confidence/depression/having a positive outlook on life and expectations for the future”^a^
Intra-operative organ injury	6 (75.0)	1 (12.5)	1 (12.5)	In	Retain item
Intra-abdominal abscess	2 (25.0)	4 (50.0)	2 (25.0)	Unsure	Voted out
Septicaemia	5 (62.5)	2 (25.0)	1 (12.5)	Unsure	Voted out
Bleeding problems—includes intra-abdominal, gastrointestinal, and staple line bleeding	3 (37.5)	2 (25.0)	3 (37.5)	Unsure	Voted out
Gastric band problems	3 (37.5)	3 (37.5)	2 (25.0)	Unsure	Retain “leaks, fistulas, strictures, and ulcers at anastomosis/gastric band problems”^a^
Port problems	1 (12.5)	5 (62.5)	2 (25.0)	Unsure	Retain “leaks, fistulas, strictures, and ulcers at anastomosis/gastric band problems”^a^
Internal hernia	7 (87.5)	1 (12.5)	0 (0.0)	In	Retain item
Needing to go to ITU for ventilation	1 (12.5)	3 (37.5)	4 (50.0)	Unsure	Voted out
Cardiac problems due to surgery	4 (50.0)	0 (0.0)	4 (50.0)	Unsure	Voted out
Venous thromboembolism due to surgery	4 (50.0)	0 (0.0)	4 (50.0)	Unsure	Voted out
Stroke due to surgery	3 (37.5)	2 (25.0)	3 (37.5)	Unsure	Voted out
Renal failure due to surgery	5 (62.5)	2 (25.0)	1 (12.5)	Unsure	Voted out
Dysphagia/regurgitation	6 (75.0)	0 (0.0)	2 (25.0)	In	Retain item
Micronutrient status	5 (62.5)	3 (37.5)	0 (0.0)	Unsure	Retain item
Readmission rates	5 (62.5)	1 (12.5)	2 (25.0)	Unsure	Voted out

ITU = Intensive Treatment Unit.

^a^ Item retained and merged with at least two other items.

**Table 4:** Results of the health professional consensus meeting.

	Number of professionals				
	(out of 33) voting (%)				
Item	Item In	Unsure	Item Out	Initial views	Final decision
Diabetes status	Definitely in COS—not voted on				Retain item
Mortality (30-day or long-term)	Definitely in COS—not voted on				Retain item
Leaks, fistulas, strictures, and ulcerations at anastomosis	Definitely in COS—not voted on				Retain “technical complications of the specific operation” and “any re-operation/re-intervention and its classification of severity”^a^
Weight	Definitely in COS—not voted on				Retain item
Hypertension	17 (51.5)	4 (12.1)	12 (36.4)	Unsure	Voted out
Cardiovascular risk	23 (69.7)	2 (6.1)	8 (24.2)	In	Retain item
Dyslipidaemia	7 (21.2)	5 (15.2)	21 (63.6)	Unsure	Voted out
Obstructive sleep apnoea	16 (48.5)	3 (9.1)	14 (42.4)	Unsure	Voted out
Joint disease	6 (18.2)	2 (6.1)	25 (75.8)	Out	Voted out
Ability to carry out usual activities	14 (42.4)	1 (3.0)	18 (54.5)	Unsure	Retain “overall quality of life”^a^
Mobility	24 (72.7)	2 (6.1)	7 (21.2)	In	Retain “overall quality of life”^a^
Ability to do your work, or to take up work	13 (39.4)	4 (12.1)	16 (48.5)	Unsure	Retain “overall quality of life”^a^
Having a healthy / balanced eating pattern	11 (33.3)	3 (9.1)	19 (57.6)	Unsure	Retain “overall quality of life”^a^
Ability to stop eating when feeling full	11 (33.3)	3 (9.1)	19 (57.6)	Unsure	Retain “overall quality of life”^a^
Self-esteem and self-confidence	15 (45.5)	6 (18.2)	12 (36.4)	Unsure	Retain “overall quality of life”^a^
Depression	10 (30.3)	3 (9.1)	20 (60.6)	Unsure	Retain “overall quality of life”^a^
Anxiety	1 (3.0)	2 (6.1)	30 (90.9)	Out	Retain “overall quality of life”^a^
Feeling able to live a “normal” life	12 (36.4)	6 (18.2)	15 (45.5)	Unsure	Retain “overall quality of life”^a^
Feeling in control of health and well-being	13 (39.4)	3 (9.1)	17 (51.5)	Unsure	Retain “overall quality of life”^a^
Having a positive outlook on life and expectations for the future	8 (24.2)	3 (9.1)	22 (66.7)	Unsure	Retain “overall quality of life”^a^
Intra-operative organ injury	3 (9.1)	2 (6.1)	28 (84.8)	Out	Retain “technical complications of the specific operation” and “any re-operation/re-intervention and its classification of severity”^a^
Intra-abdominal abscess	6 (18.2)	3 (9.1)	24 (72.7)	Out	Retain “technical complications of the specific operation” and “any re-operation/re-intervention and its classification of severity”^a^
Septicaemia	10 (30.3)	2 (6.1)	21 (63.6)	Unsure	Retain “any re-operation/re-intervention and its classification of severity”^a^
Bleeding problems—includes intra-abdominal, gastrointestinal and staple line bleeding	17 (51.5)	2 (6.1)	14 (42.4)	Unsure	Retain “technical complications of the specific operation” and “any re-operation/re-intervention and its classification of severity”^a^
Gastric band problems	22 (66.7)	3 (9.1)	8 (24.2)	Unsure	Retain “technical complications of the specific operation” and “any re-operation/re-intervention and its classification of severity”^a^
Port problems	11 (33.3)	1 (3.0)	21 (63.6)	Unsure	Retain “technical complications of the specific operation” and “any re-operation/re-intervention and its classification of severity”^a^
Internal hernia	20 (60.6)	0 (0.0)	13 (39.4)	Unsure	Retain “technical complications of the specific operation” and “any re-operation/re-intervention and its classification of severity”^a^
Needing to go to ITU for ventilation	8 (24.2)	3 (9.1)	22 (66.7)	Unsure	Retain “any re-operation/re-intervention and its classification of severity”^a^
Cardiac problems due to surgery	4 (12.1)	2 (6.1)	27 (81.8)	Out	Retain “any re-operation/re-intervention and its classification of severity”^a^
Venous thromboembolism due to surgery	15 (45.5)	2 (6.1)	16 (48.5)	Unsure	Retain “any re-operation/re-intervention and its classification of severity”^a^
Stroke due to surgery	2 (6.1)	2 (6.1)	29 (87.9)	Out	Retain “any re-operation/re-intervention and its classification of severity”^a^
Renal failure due to surgery	2 (6.1)	1 (3.0)	30 (90.9)	Out	Retain “any re-operation/re-intervention and its classification of severity”^a^
Dysphagia/regurgitation	26 (78.8)	0 (0.0)	7 (21.2)	In	Retain item
Micronutrient status	27 (81.8)	0 (0.0)	6 (18.2)	In	Retain item
Readmission rates	24 (72.7)	2 (6.1)	7 (21.2)	In	Retain “any re-operation/re-intervention and its classification of severity”^a^

ITU = Intensive Treatment Unit.

^a^ Item retained and merged with at least two other items.

After the initial round of anonymised voting at the patients’ meeting, six items were voted “In,” three “Out,” and 22 “Unsure” (Table 3). At the professionals’ meeting, five were voted “In,” seven “Out,” and 19 “Unsure” (Table 4). “Unsure” items underwent further discussion and voting. Extensive discussion in meetings revealed that some items overlapped in content and meaning. Thus, some were merged into a single item. For example, at the professionals’ meeting, the consensus was that the ten items relating to quality of life (QOL) (e.g., “mobility,” “self-esteem and self-confidence”) should be combined into a single item, “overall quality of life.” Professionals indicated that they would have liked to include all ten QOL items (which would have meant 18 items in the final COS). However, they were aware of the importance of limiting the final COS for it to be feasible to use in future trials. Therefore, the consensus was to include one QOL item that would encompass all of the more specific items. Similarly, items relating to potential complications of surgery were combined into two items, “technical complications of the specific operation” and “any re-operation/re-intervention and its classification of severity.” After voting and discussion, an additional six items were included by patients and four items by professionals. Thus, the final COSs agreed by patients and professionals included 12 and nine items, respectively (Table 5). When comparing COSs, all 12 items included in the patient COS were represented in the health professional COS, as professionals merged four items included by patients as “overall quality of life.” The only item included by health professionals that was not included by patients was “cardiovascular risk.” Thus, the final COS includes nine items (Table 5).

**Table 5:** Comparison of health professional and patient final COSs.

	Health professional COS^a^		Equivalent items included in patient COS
1	Weight	1	Weight
2	Diabetes status	2	Diabetes status
3	Cardiovascular risk		Not included by patients
4	Overall quality of life	3–6	Mobility/ability to carry out usual activities/living a normal life
			Ability to do your work, or to take up work
			Feeling in control
			Self-esteem and self-confidence/depression/having a positive outlook on life and expectations for the future
5	Mortality (30-day or long-term)	7	Mortality (30-day or long-term)
6	Technical complications of the specific operation	8–10	Leaks, fistulas, strictures, and ulcers at anastomosis/gastric band problems
			Intra-operative organ injury
			Internal hernia
7	Any re-operation/re-intervention and its classification of severity	8, 10	Leaks, fistulas, strictures, and ulcers at anastomosis/gastric band problems
			Internal hernia
8	Dysphagia/regurgitation	11	Dysphagia/regurgitation
9	Micronutrient status	12	Micronutrient status

^a^ The final COS, as it also includes all the items in the patient COS.

Items 1–4 relate to potential benefits of the surgery, and 5–9 relate to potential complications of the surgery.

## Discussion

This study has developed a COS to use in studies of bariatric and metabolic surgery. A wide range of sources, including the literature and patient interviews, were used to inform a prioritisation exercise. This was undertaken with over 250 health professionals and patients to identify the outcomes of greatest importance. The final core set consists of nine outcomes important to different professionals and patients, including weight, diabetes, cardiovascular risk, QOL, and potential risks of the surgery. It is now recommended that researchers use the COS to inform the selection of measures used in future studies evaluating bariatric surgery.

To our knowledge, this is the first study to develop a COS for bariatric surgery including professionals’ and patients’ views. The authors of the Cochrane review of bariatric surgery noted particular problems with the heterogeneity of surgical complications reported across studies and specified that mortality and re-operation rates should be reported in all future studies [3]. The authors suspected that outcome reporting bias was particularly a problem for QOL and diabetes outcomes [3]. Therefore, it may be particularly important that these form part of the minimum COS. The COS developed in this study included the outcomes “diabetes status,” “overall quality of life,” “mortality (30-day and/or long-term),” “any re-operation/re-intervention and its classification of severity,” and thus includes all outcomes specified in the Cochrane review.

In 2004, a COS for obesity in general was published based on the International Classification of Functioning, Disability, and Health (ICF) checklist [26]. This was developed from preliminary work to develop a COS for chronic conditions in general, which included systematic reviews, a Delphi survey of health professionals working with patients with chronic conditions, and the administration (by health professionals) of the ICF checklist to patients with a range of chronic conditions [27–29]. The COS for obesity was then finalised in a consensus meeting with health professionals working in obesity and included nine items: “energy and drive,” “weight maintenance,” “general metabolic functions,” “handling stress and other psychological demands,” “walking,” “moving around,” “looking after one’s health,” “products or substances for personal consumption,” and “immediate family” [26]. The COS developed by Stucki et al. is not specific for different obesity treatments, like bariatric surgery. In comparison with our COS, the item “overall quality of life” may encompass the majority of items in their brief COS. An additional issue with the obesity COS proposed by Stucki et al. is the lack of patient input, and participating professionals were mainly physicians, with limited numbers of other health professionals [26,27]. The main reasons for including patients’ views are to ensure that benefits as well as risks of surgery are included and to keep outcomes patient centred and relevant to pragmatic trials and health services provision [30].

This study is novel and was conducted using appropriate methodology with key stakeholders, including patients, to develop a COS for bariatric surgery. However, there are some methodological limitations. There were low response rates to round 1 of the Delphi survey, which suggests that the use of questionnaires may not have appealed to all stakeholders. However, the use of a Delphi survey was felt to be the most appropriate method, as it allowed a much larger number of professionals and patients to participate than purely face-to-face methods would have, and retention rates in rounds 2 and 3 of the survey were good. A maximum variation sampling strategy was used to ensure that all predefined stakeholder groups were sampled and representative of patients undergoing surgery and relevant health professionals. It was a strength that our patient participants had a mean time since surgery of 3.5 years, as they had experience of living with the outcomes of surgery in the long term after the initial “honeymoon” phase had worn off [31]. We recognise that eight patients was a low number of participants in the consensus meeting. However, their views about which outcomes to include in the COS were supported by the professionals’ views, as well as our own experience of issues raised by patients in clinical practice. “Cardiovascular risk” is the only outcome in the COS that was included by professionals but not patients. It may be that the future “risk” of cardiovascular problems was not something patients could easily conceptualise; however, it was more of a priority for professionals who regularly see patients with cardiovascular complications. The main limitation of this study was that it was based only in the UK, although a few professionals from other countries participated.

One next essential step is to undertake validation of the COS internationally and/or develop the core outcome measures working with the international community. This could involve undertaking consensus meetings with professionals and patients in other countries. The OMERACT (Outcome Measures in Rheumatology) group have published guidance on the selection of appropriate measures for COSs [32]. Further consensus methods will determine how technical complications of the specific operations and re-operations/re-interventions should be defined, as well as the key components of QOL. Literature reviews will be undertaken to generate a list of available measurement instruments, and some instruments may need to be developed where none are available. Where more than one instrument already exists, the COSMIN (COnsensus-based Standards for the selection of health Measurement INstruments) checklist may help with the selection of the most appropriate instrument [33]. This additional work will be crucial for the COS to gain widespread acceptance and use.

This study has used high-quality methods to develop a COS for studies evaluating bariatric and metabolic surgery. Its widespread adoption by the bariatric surgery community will improve the quality of outcome data from research studies, thus improving meta-analyses and the value of the research to clinical practice. Future work is needed to validate the COS internationally and determine how these outcomes are best measured.

## Supporting Information

S1 Table Methods to develop a COS. (DOCX)

S2 Table COS-STAR Statement completed checklist. (DOCX)

S3 Table Item wording for consensus meetings. (DOCX)

S4 Table The percentage of patients and health professionals rating each item 8–9 in rounds 1 and 2 of the survey, including items carried forward to round 3. (DOCX)

S5 Table The percentage of patients and health professionals rating each item 8–9 in round 3 of the survey, including items taken forward to consensus meetings. (DOCX)

S1 Questionnaire Round 2 health professional questionnaire (nurse questionnaire given as example). (PDF)

S2 Questionnaire Round 2 patient questionnaire. (PDF)

S3 Questionnaire Round 3 health professional questionnaire (surgeon questionnaire given as example). (PDF)

S4 Questionnaire Round 3 patient questionnaire. (PDF)

## Abbreviations

BARIACT: BARIAtric and metabolic surgery Clinical Trials
COMET: Core Outcome Measures in Effectiveness Trials
COS: Core Outcome Set
COSMIN: COnsensus-based Standards for the selection of health Measurement Instruments
COS-STAR: Core Outcome Set-STAndards for Reporting
ICF: International Classification of Functioning, Disability and Health
OMERACT: Outcome Measures in Rheumatology
QOL: Quality of Life
RCT: Randomised Controlled Trial
SD: Standard Deviation
