# Supplementary material for: A Core Outcome Set for the Benefits and Adverse Events of Bariatric and Metabolic Surgery: The BARIACT Project
Source: PLoS Med. 2016 Nov 29;13(11):e1002187. doi: 10.1371/journal.pmed.1002187 (PMC5127500; doi:10.1371/journal.pmed.1002187)
Supplement: S1 Table — (DOCX) [file pmed.1002187.s001.docx]

**S1 Table: Methods to develop a COS**

| **Phase 1** | **Creation of a comprehensive list of outcomes to inform a questionnaire** |
| --- | --- |
| a. | Systematic literature reviews to identify clinical outcomes and patient-reported outcomes |
| b. | Semi-structured interviews to elicit additional outcomes of importance to patients |
| c. | Outcomes from a. and b. were combined to create a long list of outcomes of bariatric surgery |
| d. | Outcomes were mapped into health domains^a^ and overlapping outcomes removed by expert health professionals and researchers, with patient feedback. |
| e. | The final list of outcomes and domains is used to develop items for a questionnaire |
| **Phase 2** | **Prioritization of outcomes in a three-round questionnaire survey** |
| a. | Round 1: Stakeholders (patients and health professionals^b^) are recruited and asked to rate the importance of each item on the questionnaire. |
| b. | Round 2: Results of round 1 are fed back to stakeholders in a second round of the survey (Delphi methodology) and stakeholders re-rate the importance of each item, taking into account this feedback |
| c. | Round 2 is analysed using predefined criteria to reduce the list of items taken forward to next round of the survey |
| d. | Round 3: Results of round 2 are fed back to stakeholders in a third round of the survey and stakeholders are asked to re-rate the importance of each item, taking into account this feedback |
| e. | Round 3 is analysed using predefined criteria to reduce the list of items. This produces a list of ‘very important’ items to be taken forward to phase 3. |
| **Phase 3** | **Stakeholder consensus meetings** |
| a. | The list of items kept in from round 3 are presented to patients and health professionals separately, and items are voted on anonymously, with three voting options: ‘In’, ‘Out’ or ‘Unsure’ |
| b. | Voting is analysed using predefined criteria to either include or exclude items from the Core Outcome Set. Items where there is no consensus undergo discussion and further voting. |
| c. | The process produces two Core Outcome Sets, selected by patients and by professionals. These are compared and combined into one Core Outcome Set. |
