# Supplementary material for: A Core Outcome Set for the Benefits and Adverse Events of Bariatric and Metabolic Surgery: The BARIACT Project
Source: PLoS Med. 2016 Nov 29;13(11):e1002187. doi: 10.1371/journal.pmed.1002187 (PMC5127500; doi:10.1371/journal.pmed.1002187)
Supplement: S2 Table — (DOCX) [file pmed.1002187.s002.docx]

**S2 Table: COS-STAR Statement completed checklist (from Kirkham et al.^1^)**

| **SECTION/TOPIC** | **ITEM No.** | **CHECKLIST ITEM** | **OUR PAPER** |
| --- | --- | --- | --- |
| TITLE/ABSTRACT |  |  |  |
| Title | 1a | Identify in the title that the paper reports the development of a COS | See title |
| Abstract | 1b | Provide a structured summary | See abstract |
| INTRODUCTION |  |  |  |
| Background and objectives | 2a | Describe the background and explain the rationale for developing the COS | Introduction, paragraph 1 |
|  | 2b | Describe the specific objectives with reference to developing a COS | Introduction, paragraph 2 |
| Scope | 3a | Describe the health condition(s) and population(s) covered by the COS | Introduction, paragraphs 1,2 |
|  | 3b | Describe the intervention(s) covered by the COS | Introduction, paragraphs 1,2 |
|  | 3c | Describe the setting(s) in which the COS is to be applied | Introduction, paragraph 2 |
| METHODS |  |  |  |
| Protocol/Registry entry | 4 | Indicate where the COS development protocol can be accessed, if available and/or the study registration details | Methods, paragraph 1 |
| Participants | 5 | Describe the rationale for stakeholder groups involved in the COS development process, eligibility criteria for participants from each group and a description of how the individuals involved were identified | Methods, paragraph 3 (“Phase 2…”) |
| Information sources | 6a | Describe the information sources used to identify an initial list of outcomes | Methods, paragraph 2 (“Phase 1…”) |
|  | 6b | Describe how outcomes were dropped/combined, with reasons (if applicable) | Methods, paragraph 2 (“Phase 1…”) |
| Consensus process | 7 | Describe how the consensus process was undertaken | Methods, paragraph 4 (“Phase 2…”) |
| Outcome scoring | 8 | Describe how outcomes were scored and scores summarised | Methods,  paragraphs 4 (“Phase 2…”), 7 (“Phase 3…”), 8 (“Statistical analyses”) |
| Consensus definition | 9a | Describe the consensus definition | Methods, paragraph 9 (“Statistical analyses”) |
|  | 9b | Describe the procedure for determining how outcomes were included or excluded from consideration during the consensus process | Methods, paragraph 9 (“Statistical analyses”) |
| Ethics and consent | 10 | Provide a statement regarding the ethics and consent issues for the study | Methods (“Ethics statement”) |
| RESULTS |  |  |  |
| Protocol deviations | 11 | Describe any changes from the protocol (if applicable), with reasons, and a describe what impact these changes have on the results | N/A |
| Participants | 12 | Present data on the number and relevant characteristics of the people involved at all stages of COS development | Results, paragraphs 2,3 (“Phase 2”), 6 (“Phase 3”) |
| Outcomes | 13a | List all outcomes considered at the start of the consensus process | Results, paragraph 1 (“Phase 1” - citation given to our previous paper where questionnaire with all outcomes is provided) |
|  | 13b | Describe any new outcomes introduced and any outcomes dropped, with reasons, during the consensus process | Results, paragraphs 4,5 (“Phase 2”), 8,9 (“Phase 3”), Tables 1-4 |
| Core outcome set | 14 | List the outcomes in the final core outcome set | Table 5 |
| DISCUSSION |  |  |  |
| Limitations | 15 | Discuss any limitations in the COS development process | Discussion, paragraph 4 |
| Conclusions | 16 | Provide an interpretation of the final COS in the context of other evidence, and implications for future research | Discussion, paragraphs 2,3,5 |
| OTHER INFORMATION |  |  |  |
| Funding | 17 | Describe sources of funding, role of funders | See funding information |
| Conflicts of interest | 18 | Describe any conflicts of interest within the study team and how these were managed | See conflict of interest statement |

^1^Kirkham JJ, Gorst S, Altman DG, Blazeby JM, Clarke M, Devane D, et al. (2016) Core Outcome Set–STAndards for Reporting: The COS-STAR Statement. PLoS Med 13(10): e1002148. doi:10.1371/journal.pmed.1002148.
