# Supplementary material for: A Core Outcome Set for the Benefits and Adverse Events of Bariatric and Metabolic Surgery: The BARIACT Project
Source: PLoS Med. 2016 Nov 29;13(11):e1002187. doi: 10.1371/journal.pmed.1002187 (PMC5127500; doi:10.1371/journal.pmed.1002187)
Supplement: S3 Table — (DOCX) [file pmed.1002187.s003.docx]

**S3 Table: Item wording for consensus meetings^a^**

|  | **Outcomes of obesity surgery definitely in the core outcome set (4)** |
| --- | --- |
|  | Improvement in diabetes |
|  | Mortality - 30 day or long-term (Death within 30 days of surgery or long-term) |
|  | Leaks, fistulas, strictures, and ulcerations at anastomosis (Problems with the new join between the two pieces of bowel) |
|  | Weight |
|  | **Outcomes of obesity surgery to be voted on (31)** |
|  | **Potential benefits of weight loss surgery** |
| 1 | Reduction in hypertension (Reduction in blood pressure) |
| 2 | Reduction in cardiovascular risk (Reduction in risk of heart problems) |
| 3 | Reduction in dyslipidaemia (Reduction in blood cholesterol) |
| 4 | Reduction in obstructive sleep apnoea (Reduction in sleep apnoea) |
| 5 | Improvement in joint disease |
| 6 | Ability to carry out usual activities |
| 7 | Improved mobility |
| 8 | Ability to accomplish work tasks or take up work (Ability to do your work, or to take up work) |
| 9 | Having a healthy / balanced eating pattern |
| 10 | Ability to stop eating when feeling full |
| 11 | Improved self-esteem and self-confidence |
| 12 | Improvement in depression |
| 13 | Reduction in anxiety |
| 14 | Feeling able to live a ‘normal’ life (Living a ‘normal’ life) |
| 15 | Feeling in control of health and well-being |
| 16 | Having a positive outlook on life and expectations for the future |
|  | **Potential complications of the surgery** |
| 17 | Intra-operative organ injury (Damage to other organs during the operation) |
| 18 | Intra-abdominal abscess (Infection inside the body due to surgery) |
| 19 | Septicaemia (Infection requiring prolonged admission to hospital) |
| 20 | Bleeding problems – includes intra-abdominal, gastrointestinal and staple line bleeding (Bleeding problems) |
| 21 | Gastric band problems |
| 22 | Port problems |
| 23 | Internal hernia (Bowel problems that need additional surgery) |
| 24 | Needing to go to ITU for ventilation (Needing to go to ITU for breathing problems post-surgery) |
| 25 | Cardiac problems due to surgery (Heart problems due to surgery) |
| 26 | Venous thromboembolism (Blood clot in the leg or lung) |
| 27 | Stroke |
| 28 | Renal failure (Kidney failure) |
| 29 | Dysphagia / regurgitation (Problems swallowing or bringing food back up) |
| 30 | Problems with micronutrient levels (Problems with vitamin and mineral levels) |
| 31 | Re-admission rates (Unexpected re-admission to hospital) |

^a^Exact wording of items used at both consensus meetings shown. Where there is alternative wording in brackets, this was used for the patient consensus meeting instead of the wording not in brackets.
