# Supplementary material for: A Core Outcome Set for the Benefits and Adverse Events of Bariatric and Metabolic Surgery: The BARIACT Project
Source: PLoS Med. 2016 Nov 29;13(11):e1002187. doi: 10.1371/journal.pmed.1002187 (PMC5127500; doi:10.1371/journal.pmed.1002187)
Supplement: S4 Table — (DOCX) [file pmed.1002187.s004.docx]

**S4 Table: The percentage of patients and health professionals rating each item 8–9 in rounds 1 and 2 of the survey, including items carried forward to round 3**

|  | **% rating item 8-9** | | | |  |
| --- | --- | --- | --- | --- | --- |
| **Item (130)^a^** | **Round 1** | | **Round 2** | | **Item carried forward to round 3?^b^** |
|  | **Patients**  **(n=90)** | **HCPs**  **(n=168)** | **Patients (n=80)** | **HCPs (n=120)** |  |
| Perioperative haemorrhage | 55.1 | 47.9 | 61.3 | 45.4 | N |
| Intra-operative organ injury | 68.2 | 54.5 | 78.8 | 62.2 | Y |
| Conversion to open surgery | 36.0 | 40.4 | 33.8 | 35.3 | N |
| Deep abscess | 68.5 | 56.0 | 76.3 | 68.1 | Y |
| Wound infection or dehiscence | 64.0 | 38.3 | 71.3 | 35.3 | Y |
| Septicaemia | 83.1 | 70.5 | 87.5 | 85.7 | Y |
| Gastrointestinal bleeding | 62.9 | 56.3 | 72.2 | 67.8 | **Y** |
| Intra-abdominal bleeding | 60.7 | 61.2 | 71.3 | 78.2 | **Y** |
| Wound bleeding / port site haematoma | 51.1 | 24.2 | 57.5 | 23.7 | N |
| Gastric fistula | 82.0 | 79.3 | 86.1 | 89.0 | **Y** |
| Anastomotic leak | 86.5 | 84.1 | 91.3 | 92.4 | **Y** |
| Bowel stenosis | 76.4 | 62.8 | 80.0 | 79.7 | Y |
| Staple line bleed | 73.0 | 56.2 | 78.8 | 77.3 | **Y** |
| Anastomotic ulceration | 71.9 | 56.1 | 76.3 | 67.2 | Y |
| Band infection | 66.7 | 71.5 | 78.7 | 84.9 | **Y** |
| Port erosion or revisions | 55.1 | 52.1 | 70.7 | 61.3 | **Y** |
| Port infection | 62.8 | 52.1 | 73.3 | 67.2 | **Y** |
| Band erosion | 78.2 | 79.4 | 84.0 | 94.9 | **Y** |
| Band slippage | 75.6 | 78.2 | 82.7 | 94.9 | Y |
| Intolerance of the band | 59.0 | 49.7 | 65.3 | 50.8 | N |
| Pouch dilation | 50.0 | 32.3 | 58.7 | 39.8 | N |
| Iatrogenic injury (to the band) | 59.0 | 47.0 | 65.3 | 43.2 | N |
| Port malfunction | 66.7 | 40.0 | 73.3 | 41.5 | **Y** |
| Band revisions | 59.2 | 67.9 | 74.7 | 78.8 | **Y** |
| Internal hernia | 66.7 | 74.5 | 78.5 | 89.8 | Y |
| External hernia, incisional | 44.8 | 26.1 | 53.2 | 29.7 | N |
| Adhesional obstruction | 69.0 | 47.3 | 71.8 | 49.2 | Y |
| Ileus | 48.3 | 27.9 | 47.4 | 30.5 | N |
| Atelectasis | 65.9 | 29.7 | 65.8 | 27.1 | N |
| Requirement for ventilation | 64.8 | 55.8 | 73.4 | 62.7 | Y |
| Lower respiratory tract infection | 50.0 | 23.6 | 54.4 | 19.5 | N |
| Ischaemic/coronary heart disease (as a complication of surgery) | 73.6 | 41.5 | 77.2 | 41.5 | **Y** |
| Arrhythmia (as a complication of surgery) | 63.6 | 26.2 | 71.8 | 21.2 | **Y** |
| Venous thromboembolism | 76.1 | 64.0 | 84.8 | 79.7 | Y |
| Stroke (as a complication of surgery) | 83.0 | 47.6 | 83.5 | 57.6 | Y |
| Renal failure (as a complication of surgery) | 80.5 | 47.0 | 81.0 | 49.2 | Y |
| Urinary tract infection (as a complication of surgery) | 44.3 | 12.2 | 51.3 | 9.3 | N |
| Incontinence (as a complication of surgery) | 53.4 | 12.8 | 55.7 | 9.3 | N |
| Perioperative mortality | 84.1 | 86.7 | 92.4 | 94.9 | **Y** |
| In hospital mortality | 83.0 | 85.5 | 91.1 | 96.6 | **Y** |
| ≤30 day mortality | 83.0 | 86.1 | 91.1 | 94.8 | **Y** |
| >30 day mortality | 80.7 | 74.1 | 87.3 | 89.7 | Y |
| Weight | 58.4 | 87.4 | 62.0 | 92.4 | **Y^c^** |
| Body dimensions (waist and hip measurements) | 44.8 | 38.6 | 36.7 | 35.6 | N |
| Body mass index | 49.4 | 76.6 | 53.2 | 86.4 | **Y^c^** |
| Improvement in arrhythmia | 62.1 | 36.1 | 67.5 | 26.3 | N |
| Reduction in hypertension | 69.7 | 76.0 | 87.5 | 84.7 | Y |
| Reduction in cardiovascular risk | 71.1 | 59.9 | 88.8 | 77.1 | Y |
| Improvement in diabetes | 82.2 | 89.8 | 92.5 | 98.3 | Y |
| Reduction in dyslipidaemia | 61.1 | 65.3 | 70.9 | 85.6 | Y |
| Reduction in obstructive sleep apnoea | 65.2 | 80.2 | 84.8 | 90.8 | Y |
| Improvement in joint disease | 65.6 | 55.7 | 86.8 | 72.9 | Y |
| Hair loss | 33.7 | 22.3 | 24.1 | 10.9 | N |
| Problems hearing | 36.0 | 6.0 | 25.3 | 2.5 | N |
| Problems with gums or teeth | 37.1 | 12.7 | 32.9 | 8.4 | N |
| Problems with vision | 44.9 | 22.9 | 45.6 | 13.4 | N |
| Oedema | 41.6 | 15.2 | 41.0 | 10.9 | N |
| Skin problems or irritations | 44.9 | 29.7 | 38.0 | 16.0 | N |
| Peripheral neuropathy/paraesthesia | 55.1 | 34.3 | 54.4 | 37.0 | N |
| Breathlessness | 51.7 | 41.6 | 64.6 | 37.8 | N |
| Flatulence | 38.2 | 18.8 | 39.2 | 13.4 | N |
| Constipation | 41.6 | 22.4 | 39.2 | 16.8 | N |
| Diarrhoea | 37.1 | 27.3 | 39.7 | 21.8 | N |
| Pain or discomfort in the body | 48.3 | 22.9 | 41.8 | 15.1 | N |
| Feeling hot or sweaty | 28.1 | 16.9 | 25.3 | 7.5 | N |
| Feeling light-headed or dizzy | 37.5 | 27.1 | 35.4 | 11.7 | N |
| Nausea | 42.7 | 35.2 | 39.2 | 25.8 | N |
| Numbness or tingling in the body | 42.7 | 22.9 | 40.5 | 15.0 | N |
| Reflux | 36.0 | 42.8 | 38.0 | 35.0 | N |
| Dysphagia/regurgitation | 49.4 | 63.3 | 65.8 | 73.3 | Y |
| Urinary incontinence | 42.7 | 15.1 | 42.3 | 10.8 | N |
| Physical fatigue | 45.5 | 35.2 | 49.4 | 23.3 | N |
| Pain or discomfort in the stomach area | 52.8 | 31.9 | 55.1 | 29.2 | N |
| Vitamin levels | 67.0 | 69.7 | 67.1 | 85.8 | **Y** |
| Energy (calorie) intake | 40.9 | 36.7 | 39.2 | 31.7 | N |
| Mineral levels | 53.4 | 65.9 | 55.7 | 80.8 | **Y** |
| Length of hospital stay | 29.2 | 54.8 | 39.2 | 51.7 | N |
| Operative time | 22.7 | 28.9 | 25.3 | 19.2 | N |
| Re-admission rates | 51.7 | 76.5 | 64.6 | 79.2 | Y |
| Ability to carry out usual activities | 58.4 | 64.7 | 73.4 | 81.7 | Y |
| Ability to shop for clothes that fit | 46.1 | 20.4 | 49.4 | 21.7 | N |
| Ability to fit into spaces in public places | 56.8 | 27.5 | 62.0 | 30.8 | N |
| Mobility | 71.9 | 65.9 | 82.3 | 78.3 | Y |
| Fitness | 61.8 | 44.9 | 73.4 | 45.8 | Y |
| Ability to participate in, and enjoy physical activities | 53.9 | 47.3 | 65.8 | 55.0 | N |
| Ability to accomplish work tasks, or to take up work | 59.6 | 70.1 | 75.9 | 82.5 | Y |
| Satisfaction and recognition at work | 33.7 | 35.3 | 48.7 | 30.8 | N |
| Relationships with work colleagues | 34.5 | 27.5 | 39.7 | 25.8 | N |
| Feeling satisfied and confident with one's body | 65.2 | 51.5 | 70.9 | 59.7 | **Y** |
| Feeling in control of weight and appearance | 68.5 | 52.1 | 77.2 | 59.7 | Y |
| Feeling like the mind and body are in tune with each other | 57.3 | 31.1 | 62.0 | 28.6 | N |
| Excess skin or skin folds following weight loss | 70.8 | 46.7 | 69.6 | 52.1 | Y^d^ |
| Having a healthy/balanced eating pattern | 71.9 | 59.0 | 77.2 | 73.9 | Y |
| Being able to recognise hunger feelings | 56.2 | 51.8 | 65.8 | 56.3 | N |
| Being able to stop eating when feeling full | 76.4 | 61.4 | 81.0 | 72.3 | Y |
| Time spent thinking about food | 41.4 | 32.9 | 35.4 | 36.1 | N |
| Eating for emotional reasons | 50.6 | 47.9 | 46.8 | 51.3 | N |
| Feeling guilty or upset after eating | 44.9 | 39.8 | 46.8 | 43.7 | N |
| Eating in secret | 52.8 | 49.4 | 57.0 | 52.1 | N |
| Eating differently in social situations than normal | 48.3 | 43.1 | 50.0 | 36.1 | N |
| How individuals feel others perceive them | 55.1 | 34.1 | 52.6 | 34.5 | N |
| Self-esteem and self-confidence | 67.4 | 58.1 | 73.4 | 58.8 | **Y** |
| Mood swings | 47.2 | 20.4 | 39.2 | 12.6 | N |
| Stress levels | 42.7 | 24.6 | 48.1 | 16.0 | N |
| Coping | 47.2 | 32.3 | 46.8 | 24.4 | N |
| Feeling in control of emotional or psychological well-being | 56.2 | 36.5 | 63.3 | 33.6 | N |
| Depression | 65.2 | 61.7 | 69.6 | 70.6 | Y |
| Anxiety | 59.6 | 55.1 | 65.8 | 66.4 | Y^d^ |
| Hostility | 51.7 | 34.7 | 48.1 | 30.5 | N |
| Fears or phobias | 29.2 | 19.2 | 31.6 | 17.6 | N |
| Suicidal thoughts | 55.1 | 56.9 | 64.6 | 73.9 | Y |
| Other addictive behaviours | 46.5 | 62.7 | 58.2 | 68.9 | Y^d^ |
| Interest in and enjoyment of sex | 40.4 | 29.3 | 43.0 | 26.9 | N |
| Ability to physically participate in sex | 44.9 | 29.9 | 48.1 | 26.1 | N |
| Sexual confidence | 46.1 | 21.0 | 51.9 | 20.2 | N |
| Sexual attention from others | 37.1 | 16.3 | 39.7 | 14.3 | N |
| Ability to fall asleep at night | 61.8 | 31.7 | 65.8 | 33.6 | N |
| Overall quality of sleep | 67.4 | 42.5 | 77.2 | 47.9 | Y |
| Sleepiness during the day | 46.1 | 41.3 | 45.6 | 42.0 | N |
| Snoring which affects others | 51.7 | 32.3 | 54.4 | 30.8 | N |
| Relationship with partner/spouse | 62.8 | 45.5 | 61.0 | 45.0 | **Y**^d^ |
| Relationship with, and/or ability to care for children | 59.8 | 51.5 | 67.9 | 54.2 | **Y**^d^ |
| Relationship with other family members | 44.9 | 28.1 | 53.2 | 26.7 | N |
| Relationship with friends | 46.1 | 25.1 | 44.3 | 22.5 | **Y**^d^ |
| Treatment from people in wider society | 38.2 | 25.9 | 36.7 | 21.7 | N |
| Confidence to participate in social activities | 51.7 | 37.1 | 57.0 | 35.8 | N |
| Feeling in control of social life | 50.6 | 33.5 | 50.0 | 31.7 | N |
| Normality | 76.4 | 54.5 | 81.0 | 71.7 | Y |
| Feeling in control of health and well-being | 72.7 | 47.2 | 79.7 | 63.0 | Y |
| Having a positive outlook on life and expectations for the future | 75.3 | 53.3 | 84.8 | 65.8 | Y |

HCPs = Healthcare professionals

^a^Item names are technical terms – see questionnaire for full item wording (S1 Text)

^b^Y=Yes (carried forward to round 3), N=No (not carried forward to round 3). Items were carried forward if rated 8 or 9 by ≥70% of either professionals or patients in round 2. Items in bold and of the same colour were merged for the round 3 questionnaire

^c^These items were merged but then used to create two new items: ‘Reduction in weight’ and ‘maintaining weight loss/preventing weight regain’

^d^Borderline items that did not reach 70% rated 8-9 but were decided to be kept in by team
