# Supplementary material for: A Core Outcome Set for the Benefits and Adverse Events of Bariatric and Metabolic Surgery: The BARIACT Project
Source: PLoS Med. 2016 Nov 29;13(11):e1002187. doi: 10.1371/journal.pmed.1002187 (PMC5127500; doi:10.1371/journal.pmed.1002187)
Supplement: S5 Table — (DOCX) [file pmed.1002187.s005.docx]

**S5 Table: The percentage of patients and health professionals rating each item 8–9 in round 3 of the survey, including items taken forward to consensus meetings**

| **Item (49)^a^** | **% rating item 8-9** | | **Item carried forward to consensus meetings?^b^** |
| --- | --- | --- | --- |
|  | **Patients (n=71)** | **HCPs (n=102)** |  |
| Intra-operative organ injury | 77.5 | 55.9 | Y |
| Deep abscess | 78.9 | 61.8 | Y |
| Wound infection or dehiscence | 56.3 | 21.6 | N |
| Septicaemia | 88.7 | 82.4 | Y |
| Intra-abdominal bleeding / gastrointestinal bleeding / staple line bleed | 80.3 | 78.4 | Y |
| Anastomotic leak / gastric fistula | 91.6 | 90.2 | **Y** |
| Bowel stricture | 83.1 | 67.7 | **Y** |
| Anastomotic ulceration | 78.9 | 52.9 | **Y** |
| Band infection, erosion and revisions | 84.4 | 84.3 | **Y** |
| Port malfunction / revisions / infection | 79.7 | 58.8 | Y |
| Band slippage | 85.9 | 88.1 | **Y** |
| Internal hernia | 82.9 | 81.4 | Y |
| Adhesional obstruction | 68.6 | 40.2 | N |
| Requirement for ventilation | 73.2 | 48.5 | Y |
| Angina / myocardial infarction / arrhythmia (as a complication of surgery) | 71.8 | 31.7 | Y |
| Venous thromboembolism | 83.1 | 77.2 | Y |
| Stroke (as a complication of surgery) | 84.5 | 59.4 | Y |
| Renal failure (as a complication of surgery) | 77.5 | 37.6 | Y |
| ≤30 day mortality | 90.1 | 94.1 | **Y** |
| >30 day mortality | 90.1 | 86.3 | **Y** |
| Dysphagia/regurgitation | 70.4 | 57.8 | Y |
| Micronutrient levels | 70.4 | 67.7 | Y |
| Re-admission rates | 73.2 | 72.6 | Y |
| Reduction in weight | 78.6 | 88.1 | **Y** |
| Weight maintenance | 87.1 | 89.1 | **Y** |
| Reduction in hypertension | 81.4 | 74.3 | Y |
| Reduction in cardiovascular risk | 84.3 | 70.0 | Y |
| Improvement in diabetes | 87.1 | 96.0 | Y |
| Reduction in dyslipidaemia | 72.9 | 64.0 | Y |
| Reduction in obstructive sleep apnoea | 84.3 | 77.2 | Y |
| Improvement in joint disease | 81.4 | 61.0 | Y |
| Ability to carry out usual activities | 81.7 | 67.7 | Y |
| Mobility | 87.3 | 75.5 | Y |
| Fitness | 65.2 | 31.4 | N |
| Ability to accomplish work tasks, or to take up work | 71.8 | 66.7 | Y |
| Feeling in control of weight and appearance | 77.5 | 50.0 | **Y** |
| Excess skin or skin folds following weight loss | 64.8 | 38.2 | N |
| Having a healthy/balanced eating pattern | 73.2 | 63.7 | Y |
| Ability to stop eating when feeling full | 83.1 | 59.8 | Y |
| Self-esteem and self-confidence | 83.1 | 68.3 | Y |
| Depression | 74.7 | 58.4 | Y |
| Anxiety | 73.2 | 48.5 | Y |
| Suicidal thoughts | 64.8 | 59.8 | N |
| Other addictive behaviours | 52.1 | 60.8 | N |
| Overall quality of sleep | 63.4 | 33.3 | N |
| Relationship with partner/spouse, friends, and/or ability to care for children | 57.8 | 37.3 | N |
| Normality | 84.3 | 65.7 | Y |
| Feeling in control of health and well-being | 84.5 | 60.8 | Y |
| Having a positive outlook on life and expectations for the future | 87.3 | 55.9 | Y |

HCPs = Healthcare professionals

^a^Item names are technical terms – see questionnaire for full item wording (S3 Text)

^b^Y=Yes (carried forward to consensus meetings), N=No (not carried forward to consensus meetings). Items were carried forward if rated 8 or 9 by ≥70% of either professionals or patients in round 3. Items in bold and of the same colour were merged for the consensus meetings.
