# Supplementary material for: A Core Outcome Set for the Benefits and Adverse Events of Bariatric and Metabolic Surgery: The BARIACT Project
Source: PLoS Med. 2016 Nov 29;13(11):e1002187. doi: 10.1371/journal.pmed.1002187 (PMC5127500; doi:10.1371/journal.pmed.1002187)
Supplement: S1 Questionnaire — (PDF) [file pmed.1002187.s006.pdf]

Developing a core outcome set for studies in weight loss surgery

Round two questionnaire

ID

Please answer all the questions yourself. The information that you provide will remain anonymous.

When you have completed the questionnaire, please return it in the stamped addressed envelope.

ConDuCT-II Hub

D

ID

SECTION 1 Short and long term complications

This section lists events that may occur during, or after, weight loss surgery. Please note, these are only possibilities and do not occur in everyone. The words in brackets are the medical terminology. Please rate how important you think it is that the following events are measured in studies of weight loss surgery and circle the number that best represents your opinion.

HCPs = Healthcare professionals.

Average previous ratings

		Nurses	Other HCPs	Patients	Your own	Not important				(Please circle)				Extremely important
	**For example:** Less pain when walking	4	5	6	5	1	2	3	4	5 (circled)	6	7	8	9

	**Complications that might occur during the operation**	Nurses	Other HCPs	Patients	Your own	Not important				(Please circle)				Extremely important
1	Bleeding problems, such as a collection of blood or loss of blood during the operation (peri- operative haemorrhage)					1	2	3	4	5	6	7	8	9
2	The risk of accidentally damaging other organs during the operation (organ injury)					1	2	3	4	5	6	7	8	9
3	The likelihood of needing to use a large cut/incision during surgery, rather than planned keyhole surgery (conversion to open)					1	2	3	4	5	6	7	8	9
	**Infection**													
4	Infection inside the body where the operation was carried out (deep abscess)					1	2	3	4	5	6	7	8	9
5	Problems with the wound such as infection, oozing, or failure to heal properly (wound infection or dehiscence)					1	2	3	4	5	6	7	8	9

D

ID

Average previous ratings

		Nurses	Other HCPs	Patients	Your own	Not important			(Please circle)				Extremely important	
6	Whole body infection which requires prolonged admission to hospital (septicaemia)					1	2	3	4	5	6	7	8	9
	**Haemorrhage**													
7	Bleeding problems in stomach or bowel which may cause blood to be seen in bowel movements (gastrointestinal bleeding)					1	2	3	4	5	6	7	8	9
8	Bleeding inside the abdomen where the operation was carried out (intra-abdominal bleeding)					1	2	3	4	5	6	7	8	9
9	Bleeding from the wound (wound bleeding / port site haematoma)					1	2	3	4	5	6	7	8	9
	**Surgical joins between internal organs (anastomosis)**													
10	Leaking of stomach contents through a hole in the stomach (gastric fistula)					1	2	3	4	5	6	7	8	9
11	Leaking of bowel contents into the abdomen through a hole where the bowel is joined or stapled (anastomotic leak)					1	2	3	4	5	6	7	8	9
12	Abnormal narrowing of the bowel caused by scar tissue or stapling, which might cause a blockage (stenosis)					1	2	3	4	5	6	7	8	9
13	Bleeding from the internal bowel staples (staple line bleed)					1	2	3	4	5	6	7	8	9
14	Ulcers developing at the new join between the two pieces of bowel (anastomotic ulceration)					1	2	3	4	5	6	7	8	9
	**Band related complications**													
15	Infection of the gastric band (band infection)					1	2	3	4	5	6	7	8	9

D

ID

Average previous ratings

		Nurses	Other HCPs	Patients	Your own	Not important			(Please circle)				Extremely important	
16	Problems with the port, such as flipping or needing the port to be relocated (port erosion or revisions)					1	2	3	4	5	6	7	8	9
17	Infection of the port which is used to change the band size (port infection)					1	2	3	4	5	6	7	8	9
18	The gastric band eroding/growing into the stomach (band moves from outside to the inside of stomach) leading to the need for further surgery (band erosion)					1	2	3	4	5	6	7	8	9
19	The band slipping out of place and needing more surgery to correct it (band slippage)					1	2	3	4	5	6	7	8	9
20	Intolerance of the band					1	2	3	4	5	6	7	8	9
21	Pain in the stomach caused by overeating/too much food stretching the stomach above the band (pouch dilation)					1	2	3	4	5	6	7	8	9
22	Damage to the band, which might require band replacement (Iatrogenic injury [device])					1	2	3	4	5	6	7	8	9
23	Failure of the port or tube to work properly (port malfunction)					1	2	3	4	5	6	7	8	9
24	The need for further surgery to make changes to the band (band revisions)					1	2	3	4	5	6	7	8	9
	**Obstruction / hernia**													
25	Twisting or abnormal movement of the bowel or intestines, which can cause blockages, pain or nausea and may need additional surgery (internal hernia)					1	2	3	4	5	6	7	8	9
26	A lump or bulge in the skin through the scar where the surgery was performed (external hernia, incisional)					1	2	3	4	5	6	7	8	9

D

ID

Average previous ratings

		Nurses	Other HCPs	Patients	Your own	Not important			(Please circle)				Extremely important	
27	Build up of scar tissue in the bowel causing obstruction or abdominal pain (adhesional obstruction)					1	2	3	4	5	6	7	8	9
28	Vomiting or excessive bloating after the operation whilst still in hospital (ileus)					1	2	3	4	5	6	7	8	9
	**General complications of surgery**													
29	One or more areas of the lungs collapsing or not inflating properly (atelectasis)					1	2	3	4	5	6	7	8	9
30	Needing a machine to help with breathing (ventilation)					1	2	3	4	5	6	7	8	9
31	Chest infection (lower respiratory tract infection)					1	2	3	4	5	6	7	8	9
32	Heart's blood supply is blocked, or interrupted, by a build-up of fatty substances in the heart's arteries (ischaemic/coronary heart disease)					1	2	3	4	5	6	7	8	9
33	Irregular or abnormal heart beat (arrhythmia)					1	2	3	4	5	6	7	8	9
34	Blood clot in the leg or lung (venous thromboembolism)					1	2	3	4	5	6	7	8	9
35	Stroke (cerebrovascular accident)					1	2	3	4	5	6	7	8	9
36	Kidney failure (renal failure)					1	2	3	4	5	6	7	8	9
37	Bladder infection in 'wee/pee' (Urinary tract infection)					1	2	3	4	5	6	7	8	9
38	Unintentional passing of urine (incontinence)					1	2	3	4	5	6	7	8	9
	**Mortality**													
39	Risk of death during the operation (peri-operative mortality)					1	2	3	4	5	6	7	8	9

D

ID

		Average previous ratings												
		Nurses	Other HCPs	Patients	Your own	Not important			(Please circle)				Extremely important	
40	Risk of death from surgical complications whilst still in hospital (in hospital mortality)					1	2	3	4	5	6	7	8	9
41	Risk of death within a month of surgery, in hospital or at home (≤30 day mortality)					1	2	3	4	5	6	7	8	9
42	Risk of death more than a month after surgery, in hospital or at home (>30 day mortality)					1	2	3	4	5	6	7	8	9

D

ID

SECTION 2 Effectiveness of weight loss surgery

The following section lists different ways to measure the effectiveness of weight loss surgery. Please rate how important you think it is that the following information is collected to measure the impact/effectiveness of the surgery and circle the number that represents your opinion. The words in brackets are the medical terminology.

Average previous ratings

	Body measurements (anthropometry)	Nurses	Other HCPs	Patients	Your own	Not important				(Please circle)			Extremely important	
43	A measurement of weight					1	2	3	4	5	6	7	8	9
44	Body dimensions such as waist and hip measurements					1	2	3	4	5	6	7	8	9
45	Body mass index					1	2	3	4	5	6	7	8	9
	**Obesity related disease**													
46	Improvement in abnormal or irregular heartbeat (arrhythmia)					1	2	3	4	5	6	7	8	9
47	Reduction/lowering of blood pressure to a healthy level, or a reduction in blood pressure medication (hypertension)					1	2	3	4	5	6	7	8	9
48	Reduction in the chance of having heart problems in the future (adjusted cardiovascular risk)					1	2	3	4	5	6	7	8	9
49	Improvement in diabetes, diabetes no longer being present, or a reduction in diabetic medication (measure of diabetes e.g. HbA1c)					1	2	3	4	5	6	7	8	9
50	Reduction in the amount of fat and cholesterol in the blood, or a reduction in medication (measure of dyslipidaemia)					1	2	3	4	5	6	7	8	9

D

ID

Average previous ratings

		Nurses	Other HCPs	Patients	Your own	Not important				(Please circle)				Extremely important
51	Being able to breathe easily when sleeping / using a sleep mask less (obstructive sleep apnoea)					1	2	3	4	5	6	7	8	9
52	Improvement in joint disease					1	2	3	4	5	6	7	8	9

D

ID

SECTION 3 Physical signs, symptoms and other measures

The following section lists some physical signs and symptoms that some people may experience before or after weight loss surgery. It also lists some measurements that may be taken. Some of these signs and symptoms may get better after surgery. Some may get worse, or remain the same. Please note, these are only possibilities and do not occur in everyone. The words in brackets are the medical terminology. These are all measured by health professionals, and are not self-reported by the patient.

Please rate how important you think it is that the following symptoms are measured in research studies of weight loss surgery, and circle the number that best represents your opinion.

Average previous ratings

	Signs	Nurses	Other HCPs	Patients	Your own	Not important			(Please circle)				Extremely important	
53	Hair loss					1	2	3	4	5	6	7	8	9
54	Problems hearing					1	2	3	4	5	6	7	8	9
55	Problems with gums or teeth					1	2	3	4	5	6	7	8	9
56	Problems with vision					1	2	3	4	5	6	7	8	9
57	Swelling or retaining water (oedema)					1	2	3	4	5	6	7	8	9
58	Skin problems or irritations (such as rashes, sores or loose skin, ulcers)					1	2	3	4	5	6	7	8	9
59	Loss of sensation in hands and feet (peripheral neuropathy/paraesthesia)					1	2	3	4	5	6	7	8	9
	Symptoms													
60	Feeling out of breath (breathlessness)					1	2	3	4	5	6	7	8	9
61	Belching, bloating, or gas (flatulence)					1	2	3	4	5	6	7	8	9
62	Constipation or difficulty passing stool					1	2	3	4	5	6	7	8	9
63	Diarrhoea or loose bowel motion					1	2	3	4	5	6	7	8	9
64	Pain or discomfort in the body					1	2	3	4	5	6	7	8	9

D

ID

Average previous ratings

		Nurses	Other HCPs	Patients	Your own	Not important			(Please circle)				Extremely important	
65	Feeling hot or sweaty					1	2	3	4	5	6	7	8	9
66	Feeling light-headed or dizzy					1	2	3	4	5	6	7	8	9
67	Feeling sick or vomiting (nausea)					1	2	3	4	5	6	7	8	9
68	Numbness or tingling in the body					1	2	3	4	5	6	7	8	9
69	Heartburn or acid indigestion (reflux)					1	2	3	4	5	6	7	8	9
70	Problems swallowing or bringing food back up (dysphagia/regurgitation)					1	2	3	4	5	6	7	8	9
71	Problems controlling the bladder (urinary incontinence)					1	2	3	4	5	6	7	8	9
72	Appearing physically tired or lacking in energy (physical fatigue)					1	2	3	4	5	6	7	8	9
73	Pain or discomfort in stomach area					1	2	3	4	5	6	7	8	9
	**Other Measures**													
74	A measurement of vitamin levels					1	2	3	4	5	6	7	8	9
75	How many calories patients consume (energy intake)					1	2	3	4	5	6	7	8	9
76	A measurement of mineral levels					1	2	3	4	5	6	7	8	9
77	The length of time spent in hospital after admission for surgery (length of hospital stay)					1	2	3	4	5	6	7	8	9
78	How long the operation takes (operative time)					1	2	3	4	5	6	7	8	9
79	Unexpected return to hospital for unplanned procedures or urgent review (re-admission rates)					1	2	3	4	5	6	7	8	9

D

ID

SECTION 4 Issues relating to quality of life and wellbeing after surgery

The following section lists some other areas of life that can be affected by having weight loss surgery. These issues are all patient reported, i.e. issues that are reported directly from the patient themselves and are not interpreted by clinicians or anyone else.

Please rate how important you think it is, that the following issues are measured in research studies of weight loss surgery, and circle the number that best represents your opinion.

Average previous ratings

	Activities of daily living and work/employment	Nurses	Other HCPs	Patients	Your own	Not important				(Please circle)				Extremely important
80	Being able to carry out usual activities (not related to paid employment) such as personal hygiene, housework, managing finances					1	2	3	4	5	6	7	8	9
81	Being able to shop for clothes that fit					1	2	3	4	5	6	7	8	9
82	Being able to fit into spaces in public places (e.g. fit into seats or through aisles)					1	2	3	4	5	6	7	8	9
83	Mobility (e.g. being able to walk, climb stairs, bend, cross legs, get up from chairs)					1	2	3	4	5	6	7	8	9
84	Fitness (strength and endurance)					1	2	3	4	5	6	7	8	9
85	Being able to participate in, and enjoy physical activities					1	2	3	4	5	6	7	8	9
86	Being able to accomplish work tasks, or to take up work/paid employment					1	2	3	4	5	6	7	8	9
87	Satisfaction and recognition at work (if in paid employment)					1	2	3	4	5	6	7	8	9
88	Relationships with work colleagues (if in paid employment)					1	2	3	4	5	6	7	8	9

D

ID

	Body image	Nurses	Other HCPs	Patients	Your own	Not important			(Please circle)					Extremely important
89	Feeling satisfied and confident with one's body					1	2	3	4	5	6	7	8	9
90	Feeling in control of weight and appearance					1	2	3	4	5	6	7	8	9
91	Feeling like the mind and body are in tune with each other					1	2	3	4	5	6	7	8	9
92	Excess skin or skin folds following weight loss					1	2	3	4	5	6	7	8	9
	**Eating behaviour**													
93	Having a healthy/balanced eating pattern					1	2	3	4	5	6	7	8	9
94	Being able to recognise hunger feelings					1	2	3	4	5	6	7	8	9
95	Being able to stop eating when feeling full					1	2	3	4	5	6	7	8	9
96	Time spent thinking about food					1	2	3	4	5	6	7	8	9
97	Eating for emotional reasons					1	2	3	4	5	6	7	8	9
98	Feeling guilty or upset after eating					1	2	3	4	5	6	7	8	9
99	Eating in secret					1	2	3	4	5	6	7	8	9
100	Eating differently in social situations than normal					1	2	3	4	5	6	7	8	9
	**Psychological and emotional wellbeing**													
101	How individuals feel others perceive them					1	2	3	4	5	6	7	8	9
102	Self-esteem and self-confidence (how someone perceives themself)					1	2	3	4	5	6	7	8	9

Average previous ratings: Nurses, Other HCPs, Patients, Your own

D

ID

Average previous ratings

		Nurses	Other HCPs	Patients	Your own	Not important		(Please circle)					Extremely important	
103	Mood swings					1	2	3	4	5	6	7	8	9
104	Stress levels					1	2	3	4	5	6	7	8	9
105	Coping (how someone feels with stress or difficulties)					1	2	3	4	5	6	7	8	9
106	Feeling in control of emotional or psychological wellbeing					1	2	3	4	5	6	7	8	9
	**Mental health**													
107	Depression					1	2	3	4	5	6	7	8	9
108	Anxiety					1	2	3	4	5	6	7	8	9
109	Hostility (level of anger, and ability to manage this)					1	2	3	4	5	6	7	8	9
110	Fears or phobias					1	2	3	4	5	6	7	8	9
111	Suicidal thoughts					1	2	3	4	5	6	7	8	9
112	Other addictive behaviours (e.g. alcohol, drugs, gambling, shopping)					1	2	3	4	5	6	7	8	9
	**Sex life**													
113	Interest in and enjoyment of sex					1	2	3	4	5	6	7	8	9
114	Ability to physically participate in sex					1	2	3	4	5	6	7	8	9
115	Sexual confidence					1	2	3	4	5	6	7	8	9
116	Sexual attention from others					1	2	3	4	5	6	7	8	9
	**Sleep**													
117	Ability to fall asleep at night					1	2	3	4	5	6	7	8	9
118	Overall quality of sleep					1	2	3	4	5	6	7	8	9
119	Sleepiness during the day					1	2	3	4	5	6	7	8	9

D

ID

Average previous ratings

		Nurses	Other HCPs	Patients	Your own	Not important			(Please circle)					Extremely important
120	Snoring which affects others					1	2	3	4	5	6	7	8	9
	**Social**													
121	Relationship with partner/spouse					1	2	3	4	5	6	7	8	9
122	Relationship with, and/or ability to care for, children					1	2	3	4	5	6	7	8	9
123	Relationship with other family members					1	2	3	4	5	6	7	8	9
124	Relationship with friends					1	2	3	4	5	6	7	8	9
125	Treatment from people in wider society					1	2	3	4	5	6	7	8	9
126	Having confidence to participate in social activities					1	2	3	4	5	6	7	8	9
127	Feeling in control of social life					1	2	3	4	5	6	7	8	9
	**Overall health, wellbeing and life**													
128	Normality (feeling able to live a 'normal' life)					1	2	3	4	5	6	7	8	9
129	Feeling in control of health and wellbeing					1	2	3	4	5	6	7	8	9
130	Having a positive outlook on life and expectations for the future					1	2	3	4	5	6	7	8	9

Thank you for your contribution to this round of the survey. Please now return the questionnaire in the pre-paid envelope provided.

Contact details
Freepost RTKR-UYTS-AKER
University of Bristol
School of Social & Community Medicine
Canynge Hall
39 Whatley Road
Bristol
BS8 2PS

katy.chalmers@bristol.ac.uk
