# Supplementary material for: A Core Outcome Set for the Benefits and Adverse Events of Bariatric and Metabolic Surgery: The BARIACT Project
Source: PLoS Med. 2016 Nov 29;13(11):e1002187. doi: 10.1371/journal.pmed.1002187 (PMC5127500; doi:10.1371/journal.pmed.1002187)
Supplement: S3 Questionnaire — (PDF) [file pmed.1002187.s008.pdf]

Developing a core outcome set for studies in weight loss surgery

Round three questionnaire

ID

Please answer all the questions yourself. The information that you provide will remain anonymous.

When you have completed the questionnaire, please return it in the pre-paid envelope.

ID

Completing the survey

Thank you for continuing to take part in this study. This questionnaire is much shorter than the previous two questionnaires. In this short questionnaire you will see your own rating (green column), those of surgeons (pink column), other clinicians (which includes doctors, nurses, dietitians, and psychologists) in the yellow column and patients (blue column). We are asking you to re-rate each item taking into account other people's views. We need your help to prioritise a small number of essential items. **The final items selected will be measured in all studies of weight loss surgery as a minimum, we hope this will be about 10 items.**

The information that you provide will remain anonymous. When you have completed the questionnaire, please return it in the pre-paid envelope provided. Thank you again so much for all your time and help with this survey, which will help us with future research.

B

ID

SECTION 1 Short and long term complications

This section lists events that may occur during, or after, weight loss surgery. Please note, these are only possibilities and do not occur in everyone. The words in brackets are the medical terminology. Please rate how important you think it is that the following events are measured in studies of weight loss surgery and circle the number that best represents your opinion.

Previous ratings

		Surgeons	Other clinicians	Patients	Your own	Not important				(Please circle)			Extremely important	
	**For example:** Less pain when walking	4	5	6	5	1	2	3	4	(5)	6	7	8	9

	**Complications that might occur during the operation**	Surgeons	Other HCPs	Patients	Your own	Not important				(Please circle)			Extremely important	
1	The risk of accidentally damaging other organs during the operation (organ injury)					1	2	3	4	5	6	7	8	9
	**Infection**													
2	Infection inside the body where the operation was carried out (deep abscess)					1	2	3	4	5	6	7	8	9
3	Problems with the wound such as infection, oozing, or failure to heal properly (wound infection or dehiscence)					1	2	3	4	5	6	7	8	9
4	Whole body infection which requires prolonged admission to hospital (septicaemia)					1	2	3	4	5	6	7	8	9
	**Haemorrhage**													
5	Bleeding problems inside the abdomen or within the bowel (intra-abdominal bleeding/gastrointestinal bleeding/staple line bleed)					1	2	3	4	5	6	7	8	9

B

ID	

Previous ratings

	Surgical joins between internal organs (anastomosis)	Surgeons	Other clinicians	Patients	Your own	Not important			(Please circle)					Extremely important
6	Leaking of bowel/stomach contents through a hole at the site of the operation (anastomotic leak/gastric fistula)					1	2	3	4	5	6	7	8	9
7	Abnormal narrowing of the bowel caused by scar tissue or stapling, which might cause a blockage (stricture)					1	2	3	4	5	6	7	8	9
8	Ulcers developing at the new join between the two pieces of bowel (anastomotic ulceration)					1	2	3	4	5	6	7	8	9
	**Band related complications**													
9	Problems with the gastric band such as infection or eroding/growing into the stomach. This may lead to further surgery (band infection, erosion and revisions)					1	2	3	4	5	6	7	8	9
10	Problems with the port or tubing, such as flipping, needing to be relocated, infection and/or needing further surgery (port malfunction/ revisions/infection)					1	2	3	4	5	6	7	8	9
11	The band slipping out of place and needing more surgery to correct it (band slippage)					1	2	3	4	5	6	7	8	9
	**Obstruction / hernia**													
12	Twisting or abnormal movement of the bowel or intestines, which can cause blockages, pain or nausea and may need additional surgery (internal hernia)					1	2	3	4	5	6	7	8	9
13	Build-up of scar tissue in the bowel causing obstruction or abdominal pain (adhesional obstruction)					1	2	3	4	5	6	7	8	9

B

ID

Previous ratings

		Surgeons	Other clinicians	Patients	Your own	Not important			(Please circle)				Extremely important	
	**General complications of surgery**													
14	Problems with breathing so that a machine to help with breathing is needed (ventilation)					1	2	3	4	5	6	7	8	9
15	Problems with the blood supply to the heart, causing pain/heart attack/irregular or abnormal heart beat (angina/myocardial infarction/arrhythmia)					1	2	3	4	5	6	7	8	9
16	Blood clot in the leg or lung (venous thromboembolism)					1	2	3	4	5	6	7	8	9
17	Stroke (cerebrovascular accident)					1	2	3	4	5	6	7	8	9
18	Kidney failure (renal failure)					1	2	3	4	5	6	7	8	9
	**Mortality**													
19	Death (during the operation or within 30 days of surgery)					1	2	3	4	5	6	7	8	9
20	Death (more than 30 days after surgery)					1	2	3	4	5	6	7	8	9
	**Other measures**													
21	Problems swallowing or bringing food back up (dysphagia/regurgitation)					1	2	3	4	5	6	7	8	9
22	A measurement of vitamin and mineral levels (micronutrient levels)					1	2	3	4	5	6	7	8	9
23	Unexpected return to hospital for unplanned procedures or urgent review (re-admission rates)					1	2	3	4	5	6	7	8	9

B

ID

SECTION 2 Effectiveness of weight loss surgery

The following section lists different ways to measure the effectiveness of weight loss surgery. Please rate how <u>important</u> you think it is that the following information is collected to <u>measure the impact/effectiveness</u> of the surgery and circle the number that represents your opinion. The words in brackets are the medical terminology.

Previous ratings

	Body measurements (anthropometry)	Surgeons	Other clinicians	Patients	Your own	Not important			(Please circle)				Extremely important	
24	Reduction in weight					1	2	3	4	5	6	7	8	9
25	Maintaining weight loss/preventing weight regain					1	2	3	4	5	6	7	8	9
	**Obesity related disease**													
26	Reduction/lowering of blood pressure, or a reduction in blood pressure medication (hypertension)					1	2	3	4	5	6	7	8	9
27	Reduction in the chance of having heart problems in the future (adjusted cardiovascular risk)					1	2	3	4	5	6	7	8	9
28	Improvement in diabetes, or a reduction in diabetic medication (measure of diabetes e.g. HbA1c)					1	2	3	4	5	6	7	8	9
29	Reduction in the amount of fat and cholesterol in the blood, or a reduction in medication (measure of dyslipidaemia)					1	2	3	4	5	6	7	8	9
30	Being able to breathe easily when sleeping / using a sleep mask less (obstructive sleep apnoea)					1	2	3	4	5	6	7	8	9
31	Improvement in joint disease					1	2	3	4	5	6	7	8	9

B

ID

SECTION 3 Issues relating to quality of life and wellbeing after surgery

The following section lists some other areas of life that can be affected by having weight loss surgery. These issues are all <u>patient reported,</u> i.e. issues that are reported directly from the patient themselves and are not interpreted by clinicians or anyone else.

Please rate how <u>important</u> you think it is, that the following issues are <u>measured in research studies</u> of weight loss surgery, and circle the number that best represents your opinion.

Previous ratings

	Activities of daily living and work/employment	Surgeons	Other clinicians	Patients	Your own	Not important				(Please circle)				Extremely important
32	Being able to carry out usual activities (not related to paid employment) such as personal hygiene, housework, managing finances					1	2	3	4	5	6	7	8	9
33	Mobility (e.g. being able to walk, climb stairs, bend, cross legs, get up from chairs)					1	2	3	4	5	6	7	8	9
34	Fitness (strength and endurance)					1	2	3	4	5	6	7	8	9
35	Being able to accomplish work tasks, or to take up work/paid employment					1	2	3	4	5	6	7	8	9
	**Body image**													
36	Feeling in control of weight and appearance					1	2	3	4	5	6	7	8	9
37	Excess skin or skin folds following weight loss					1	2	3	4	5	6	7	8	9
	**Eating behaviour**													
38	Having a healthy/balanced eating pattern					1	2	3	4	5	6	7	8	9

B

ID

		Surgeons	Other clinicians	Patients	Your own	Not important			(Please circle)				Extremely important	
		Previous ratings												
39	Being able to stop eating when feeling full					1	2	3	4	5	6	7	8	9
	**Psychological and emotional wellbeing**													
40	Improvement in self-esteem and self-confidence					1	2	3	4	5	6	7	8	9
	**Mental health**													
41	Depression					1	2	3	4	5	6	7	8	9
42	Anxiety					1	2	3	4	5	6	7	8	9
43	Suicidal thoughts					1	2	3	4	5	6	7	8	9
44	Other addictive behaviours (e.g. alcohol, drugs, gambling, shopping)					1	2	3	4	5	6	7	8	9
	**Sleep**													
45	Overall quality of sleep					1	2	3	4	5	6	7	8	9
	**Social**													
46	Relationship with partner/spouse, friends, and/or ability to care for children					1	2	3	4	5	6	7	8	9
	**Overall health, wellbeing and life**													
47	Normality (feeling able to live a ‘normal’ life)					1	2	3	4	5	6	7	8	9
48	Feeling in control of health and wellbeing					1	2	3	4	5	6	7	8	9
49	Having a positive outlook on life and expectations for the future					1	2	3	4	5	6	7	8	9

B

ID

SECTION 4 Top five outcomes to measure

Please write five outcomes you consider essential to measure when evaluating weight loss surgery:

1. ____

2. ____

3. ____

4. ____

5. ____

Thank you for your contribution to this round of the survey. Please now return the questionnaire in the pre-paid envelope provided.

Contact details
Katy Chalmers
FREEPOST RTKR-UYTS-AKER
School of Social and Community Medicine
Canynge Hall
39 Whatley Road
Bristol
BS8 2PS

katy.chalmers@bristol.ac.uk

This project is funded by the NIHR
